# Antibody-Directed Therapies: Toward a Durable and Tolerable Treatment Platform for CTCL

**DOI:** 10.3389/fonc.2019.00645

**Published:** 2019-07-30

**Authors:** Shaheer Khan, Ahmed Sawas

**Affiliations:** Department of Medicine, Center for Lymphoid Malignancies, Columbia University Medical Center, The New York Presbyterian Hospital, College of Physician and Surgeons, New York, NY, United States

**Keywords:** CTCL, antibody-directed therapy, brentuximab vedotin, mogamulizumab, PD-1, E777, TTI-621, AFM13

## Abstract

Cutaneous T-cell lymphomas (CTCL) are a rare group of heterogeneous disorders characterized by cutaneous involvement of monoclonal T-lymphocytes. Although indolent at early stages, CTCL can confer significant morbidity, and mortality when advanced. There is an unmet need for tolerable and durable treatments with antibodies recently gaining promise. Here we review approved systemic therapies and discuss select antibodies in development.

## Introduction

Cutaneous T-Cell lymphomas (CTCL) are a heterogeneous group of disorders that are characterized by cutaneous involvement of monoclonal T-lymphocytes. Mycosis fungoides (MF) is the most common form, accounting for ~50% of cases. Primary cutaneous CD30+ anaplastic large cell lymphoma, lymphomatoid papulosis, and Sezary syndrome (SS) constitute the other most common forms (1). There are ~2,500 new cases of MF and SS per year in the United States (2).

MF usually presents as erythematous patches that occur predominantly in sun-shielded areas including the torso and lower extremities. In about two thirds of patients, the disease is diagnosed at an early stage (stage IA-IIB) with a relatively indolent course. In patients with early stage MF, median survival can exceed 25 years (3). Progression to advanced stage (IIB-IV) occurs in 30% of MF cases. Sezary syndrome exhibits a more aggressive course in which systemic involvement of neoplastic T-lymphocytes results in leukemic involvement, exfoliative dermatitis, and lymph node enlargement. In advanced MF and SS, median survival has been described as short as 1.5 years (4).

Currently, there is no cure for CTCL and it is characterized by a chronic, relapsing course that requires repeat treatment regimens. Patients with early stage disease are treated with skin-directed therapies which include topical corticosteroids, phototherapy, topical chemotherapy, topical bexarotene, and radiotherapy. Patients with advanced stage disease are offered palliative systemic therapies to control symptoms and achieve disease control. Systemic therapies include bexarotene, Interferon-alpha, extracorporeal photopheresis, histone deacetylase inhibitors, chemotherapy, monoclonal antibodies (Mab), and allogeneic transplantation (5).

Most studies examining systemic treatments in CTCL have been small, single-arm studies with various response criteria. Most commonly, overall response rate (ORR) has been the primary endpoint, resulting in studies with little to no information on extended durability and tolerability. There remains a substantial need for treatments that are both tolerable and offer durable disease control.

Among therapies that have been studied in this setting, antibodies have recently gained prominence with the recent approval of two agents, increasing the limited number of approved systemic therapies in CTCL (see Table 1). In this review, we will discuss the approved antibodies as well as select agents in development.

**Table 1:** Approved systemic therapies.

**Class**	**Drug**	**Response rate %**	**Median DoR**	**Major toxicities**
Immunomodulatory	Extracorporeal photopheresis (6)	5	Not well-defined	Fluid shifts, hypotension, infection, anemia
Antimetabolite	Methotrexate (7)	33	15 months	mucositis, myelosuppression, hepatic, renal toxicity
Retinoid	Bexarotene (8)	45-55	10–13 months	hyperlipemia, hypercholesterolemia hypothyroidism
HDAC inhibitor	Vorinostat (9)	30	6 months	fatigue, diarrhea, nausea, thrombocytopenia, anorexia, taste abnormalities, weight loss, and muscle spasms
HDAC inhibitor	Romidepsin (10, 11)	34	13.7–15 months	nausea vomiting, fatigue, myelosuppression, QT interval changes
IL-2 Fusion Protein	Denileukin diftitox (12)	44	7.8 months	nausea, pyrexia, fatigue, rash, LFT abnormalities, vision changes, and capillary leak syndrome
Antibody-Drug conjugate	Brentuximab vedotin (13)	56	15.1 months	peripheral neuropathy, GI upset (nausea, diarrhea, vomiting), alopecia, pruritus, pyrexia, decreased appetite, and fatigue
Monoclonal antibody	Mogamulizumab (14)	28	15.5–25.5 months	infusion-related reactions, drug rash, diarrhea, and fatigue

## Approved Antibody Therapies

### Brentuximab Vedotin

Brentuximab vedotin (Bv) is a chimeric monoclonal antibody-drug conjugate consisting of an anti-CD30 antibody linked to an anti-microtubule agent, monomethyl auristatin E (MMAE). CD30 is relatively specific for activated leukocytes and certain hematological malignancies including Hodgkin lymphoma and ALCL. CD30 is variably expressed in MF and SS, with higher expression noted in 24% of patients with advanced MF (15, 16). Binding of the antibody to CD30 results in endocytosis of MMAE and cell death ensues via cell cycle arrest and apoptosis. Based on the positive results seen in the SG035-003 study, brentuximab vedotin was approved in 2011 in patients with relapsed/refractory Hodgkin lymphoma and systemic ALCL (17).

Based on the benefit seen in patients with Hodgkin lymphoma and ALCL, two phase II studies of Bv in CTCL were initiated. In the first study, 30 patients with MF or SS with variable CD30 expression were included with objective global response as the primary endpoint. Patients received Bv 1.8 mg/kg IV every 3 weeks for a maximum of 16 doses. Objective global response was seen in 21 patients (70% in all patients, 50% in SS patients), with one patient achieving a complete response (CR). There was no statistically significant difference in response rates between early or advanced stages. However, patients with a CD30 expression of <5% had a much lower likelihood of response compared to higher CD30 levels. The 12-month progression free survival (PFS) rate was 54% including 5 patients who remained on therapy at time of analysis. Median duration of response was not reported. The most common adverse events were peripheral neuropathy (66%), fatigue (47%), nausea (28%), alopecia (22%), and neutropenia (19%). Ten patients had a dose delay and/or reduction, and six patients (19%) were terminated from the study prematurely due to toxicities, with peripheral neuropathy as the most likely cause (18).

In the second phase II study, 48 patients with CTCL and lymphomatoid papulosis were treated with Bv 1.8 mg/kg IV every 3 weeks with ORR as the primary endpoint (19). An ORR of 73% with a CR rate of 35% was observed. In the MF and SS cohorts, ORR was >50% regardless of the degree of CD30 expression. Median duration of response (DoR) in all patients was 7.4 months (<1–23 months). Peripheral neuropathy was the most common side effect with grade 1 to 2 peripheral neuropathy observed in 65% of patients. Grade 3–4 events included neutropenia, nausea, chest pain, deep vein thrombosis, transaminitis, and dehydration. Dose reductions to 1.2 mg/kg were instituted due to neuropathy, transaminitis, arthralgias, and fatigue.

In the first frontline phase III study comparing a novel systemic therapy with standard therapies in CTCL, the randomized, open-label brentuximAb vedotin phase III trial for Cutaneous T-cell lymphomA aNalyZing pAtient outcomes (ALCANZA) study compared Bv to physician's choice of methotrexate or bexarotene in previously untreated CD30 positive CTCL (13). The primary endpoint was objective response lasting at least 4 months (ORR4). A total of 131 patients were randomized, with 128 patients in the intent-to-treat population and 64 patients assigned to each arm. Results demonstrated a clear benefit in ORR4 of 56.3 vs. 12.5% and in CR of 16 vs. 2%. Median PFS was also significantly longer (16.7 vs. 3.5 months). In patients with MF, the ORR4 rate was 50 vs. 10%. This benefit extended across all disease compartments including patients with skin-only disease (67.7 vs. 16.7%) and skin and other involvement (45.5 vs. 8.8%). CD30 expression ranged between 12 and 67.5% with responses seen at all levels. Median PFS in the MF arm was 15.9 vs. 3.5 months.

Overall, toxicity of Bv was comparable to standard therapies. Serious drug related adverse events (grade ≥3) were seen in 29% in both groups. Toxicities specific to the Bv arm included peripheral neuropathy (67 vs. 4%) resulting in discontinuation of treatment in 9 patients. Discontinuation due to adverse events occurred in 24% patients in the BV group vs. 8% in the PC group.

In November 2017, the FDA approved brentuximab vedotin for use in relapsed/refractory CD30+ mycoises fungoides or primary cutaneous ALCL (20).

*Brentuximab vedotin is a durable and effective targeted treatment for patients with CD30*+ *CTCL with a response rate* <*50%. Importantly, this benefit was seen across all levels of CD30 expression above 10%. Peripheral neuropathy is the most significant toxicity*.

### Mogamulizumab

Mogamulizumab is a humanized IgG1 monoclonal antibody which selectively binds to CCR4 (21). CCR4 is normally expressed on type 2 helper and regulatory (Treg) T-cells and has been found to be overexpressed on the surface of tumor cells in various T-cell malignancies, including in up to 52% of patients with different stages of MF and SS (22, 23). In addition to the stimulation of antibody-dependent cellular cytotoxicity, the suppression of Treg cells is thought to play an important role in the efficacy of mogamulizumab (24).

In a phase II study performed in Japan, 37 patients with relapsed/refractory CCR4+ peripheral T-cell lymphoma (*n* = 29) and MF (*n* = 7) received mogamulizumab at a dose of 1 mg/ kg IV once per week for 8 weeks with ORR as the primary endpoint. The ORR was 34% and median OS was 14.2 months. Amongst the MF subgroup, 2/7 patients had a response (25). Median PFS for the group was not reached, however, for the PTCL group it was 14.2 months. Based on these results, mogamulizumab was approved in Japan in 2014 for relapsed/refractory CCR4-positive peripheral T-cell lymphoma and cutaneous T-cell lymphoma (26).

Subsequently, a multi-center phase I/II study was performed in the US which enrolled 41 patients with CTCL. Patients received mogamulizumab at a dose of 1 mg/kg IV once per week for 4 weeks and then every 2 weeks until disease progression. Among 38 evaluable patients (21 MF, 17 SS), no dose-limiting toxicities were observed, and the maximum tolerated dose was not reached. In the phase II component the observed ORR was 37%. Patients with SS had a higher response rate (48%) compared with MF (28.6%). Responses were noted in all compartments in both MF and SS. Median PFS and duration of response (DOR) for the entire group were 11.4 and 10.4 months, respectively. The majority of toxicities were grade 1 or 2 with nausea, chills, infusion reaction, pyrexia, and fatigue being the most common. 7 patients (17%) developed a cutaneous skin eruption necessitating study withdrawal (27).

This led to the randomized, open-label phase 3 study comparing Mogamulizumab vs. VORinostat In patients with previously treated CTCL (MAVORIC). In this study, 372 patients with relapsed/refractory MF or SS who had failed at least one line of systemic therapy were randomized to receive 1 mg/kg of mogamulizumab IV weekly for the first 4 weeks and then every 2 weeks, or vorinostat 400 mg po daily. PFS was the primary endpoint. Results demonstrated superior PFS in the mogamulizumab arm with a median PFS of 7.7 months compared to 3.1 months in the vorinostat group. The hazard ratio for patients with MF was 0.72 (0.51–1.01) compared to 0.32 in patients with SS (0.21–0.49). Also noted was an increased ORR of 28% with mogamulizamab compared to 4.8% with vorinostat. The response rate was higher amongst patients with SS (37%) than in patients with MF (21%). Duration of response also differed, with median of 13.1 months in MF compared with 17.3 months in SS. Patients achieved responses in skin (42%), blood (68%), and lymph nodes (17%). Adverse events were higher with mogamulizumab, with the most common being infusion reaction (34%), and skin eruption (24%). Discontinuation of treatment due to drug rash occurred in 7% of patients. There were also two reported cases of Stevens-Johnson syndrome, including one that resulted in death. These reactions are thought to be the result of dramatic decreases in the number of Treg cells resulting in suppression of their immunosuppressive properties (14).

Based on the results of these studies, mogamulizumab received FDA approval in August, 2018 for previously treated patients with mycosis fungoides or Sézary syndrome (28).

*Mogamulizumab is a generally tolerable and effective therapy in CTCL with particularly high response in leukemic disease and well-demonstrated durability that is applicable to the majority of patients with CTCL. Skin toxicity remains a prominent concern and careful monitoring and assessment of skin rashes on therapy is important to distinguish toxicity against progression of disease*.

### Denileukin Diftitox

Denileukin diftitox or DAB389IL-2, is a genetically engineered IL-2 fusion toxin fusion protein designed to direct the cell-killing action of diphtheria toxin to cells which express the IL-2 receptor, of which CD25 is a component. CD25 expression occurs in approximately 50% of patients with CTCL. DAB389IL-2 binds selectively to CD25 after which it is internalized and eventually released in the cytosol where it inhibits protein synthesis and leads to cell death (29).

A phase I trial of an initial version of denileukin diftitox (DAB486IL-2) was conducted in patients with CD25 expressing hematological malignancies with demonstration of safety and tolerability and a ORR of 17% in CTCL patients, including 1 CR (30). A subsequent phase II study involving 14 patients with advanced CTCL resulted in 1 partial response (PR) and 2 patients with SS who demonstrated major cutaneous improvement without change in circulating Sezary cells (31).

A form of the fusion protein DAB389IL-2 with higher affinity to CD25 was then developed. In a phase I/II clinical trial of patients with NHL, HL, or CTCL with IL-2R expression (>20% by IHC), 35 patients received DAB389IL-2 in a dose escalation manner on days 1–5 of each 21-day cycle. Doses of <31 mcg/kg/day were well tolerated. DAB389IL-2 demonstrated significant activity in patients with CTCL with an ORR of 37%, including 5 CR. Infusion-related reactions developed in 74% of patients. Fever, chills, nausea, asthenia, and mild hypotension were noted in 50% of patients (32). Following these results, a randomized phase III multicenter trial compared two different doses of DAB389IL-2 in 73 patients with advanced stage recurrent CTCL who had received a median of 5 lines of previous therapy. Patients were randomly assigned to receive 9 or 18 mcg/kg/day IV for five consecutive days and treatment was repeated every 21 days for up to 8 cycles. Results demonstrated an ORR of 30% with 10% of patients achieving a CR and a mDOR of 4.4 months. There was no association between response and dose. Infusion reactions were the most common side effect (74%) with a significant number of patients developing vascular leak syndrome (23%), skin rash (42%), and grade 3/4 transaminitis (38%) (33).

A subsequent placebo-controlled phase III clinical trial was conducted in 144 patients with stage IA to III CD25+ CTCL, 123 of which had MF and 9 with SS (12). Patients received either 9 or 18 μg/kg/day for 5 days every 21 days. ORR was significantly higher in the 18 mcg/kg/day arm at 44 (10% CR) vs. 15.9%. Median PFS was also significantly longer compared with placebo (26.1 vs. 4 months). Median DOR was 7.8 months compared to 2.7 months. Significant reported adverse events included nausea (10%), pyrexia (11%), fatigue (12%), and capillary leak syndrome (4%). Discontinuation due to treatment was seen in 17% of patients.

DAB389IL-2 received full FDA approval in 2008 for patients with resistant and recurrent CTCL (34). Unfortunately, production of DAB389IL-2 was discontinued in 2014 due to production issues related to the bacterial expression system and it is currently not available for clinical use.

*Denileukin diftitox (Ontak) is an active and durable treatment option for patients with CD-25 positive CTCL, although currently unavailable. Given the substantial potential benefit, however, novel agents targeting the IL2-R mechanism are worthy of further development*.

## Experimental Antibody Therapies

### PD-1/PD-L1

Following the demonstration of Programmed Death-ligand 1 (PD-L1) expression in a subset of patients with MF and SS (35), a phase 2 study of pembrolizumab in 24 patients with MF (*n* = 9) and SS (*n* = 15) who were previously treated with at least 1 systemic therapy was initiated. Patients received 2 mg/kg IV every 3 weeks for up to 2 years. In preliminary results published in abstract form after a median follow up of 51 weeks, the ORR was 38% with 1 CR and 8 PR (56% RR in MF, 27% in SS). The median duration of response was 14 months and 2-year PFS rate was 69%. The toxicity profile was consistent with immune-mediated adverse events seen in other studies with one case of grade 2 pneumonitis and one case of grade 3 diarrhea (36). More recently, a phase II study assessed pembrolizumab in 18 patients with relapsed or refractory systemic T-cell lymphomas. In 13 evaluable patients (7 with PTCL-NOS, 3 with MF), the ORR was 33% with 4 patients achieving complete response. The median PFS was 3.2 months and median OS was 10.6 months. The trial was halted early after a preplanned futility analysis for PFS. Of note, 1 out of the 3 patients with MF had an ongoing complete response (37). Nivolumab has been studied in a phase Ib study in patients with relapsed or refractory hematologic malignancies, including 13 patients with MF. An ORR of 15% (2/13) was noted with both being partial responses. Median PFS was 10 weeks (38). There is an ongoing phase II study combining pembrolizumab with IFN-gamma in patients with previously treated MF or SS (NCT03063632).

*Early data suggests that immunotherapy with anti-PD1/PD-L1 agents offers a promising approach in CTCL with ongoing research combining these agents with other immunomodulatory agents in an effort to achieve a synergistic response*.

### IL-2 Fusion Toxin

After the discontinuation of denileukin diftitox, several novel IL-2 fusion proteins have been developed and are currently in pre-clinical testing. E777 is a recombinant cytotoxic fusion protein composed of diphtheria toxin fragments A and B and human IL-2. In a phase 1 open-label study in 17 patients with CTCL (13 with MF, 4 with SS) patients received doses ranging from 6 to 15 mcg/kg IV for the first 5 days of every 21-day cycle. Results of the lead-in portion demonstrated an ORR in 5 patients including 3 patients with stage IV disease (39). A phase 1 study of E7777 was conducted in Japan in 13 patients with relapsed/refractory PTCL (*n* = 10) and CTCL (*n* = 3, all with MF). Patients received 6, 12, and 9 mcg/kg IV with the same schedule. 9 mcg/kg/day was the maximum tolerated dose. Partial response was observed in 5/13 patients including 1/3 patients with MF. It was generally well tolerated at the recommended dose and with steroid pre-treatment with decreased rates of capillary leak syndrome, and infusion reaction compared to denileukin diftitox (40). There is an ongoing phase 2 study assessing the safety and efficacy of E7777 in persistent/recurrent CTCL (NCT01871727).

*E777 represents a new class of IL-2 fusion toxin agents which may offer the benefit of denileukin diftitox with an improved toxicity profile*.

### Alemtuzumab

Alemtuzumab is a humanized IgG1 antibody directed against CD52, which is expressed on most malignant lymphocytes but is not expressed on hematopoietic stem cells. Binding of the antibody results in cell death via various proposed mechanism including antibody-dependent cellular cytotoxicity, complement-mediated cell lysis, and apoptosis (41) (28). In a phase 2 study of 22 patients with advanced MF (*n* = 15) and SS (*n* = 7), patients were treated with a rapidly escalating dose regimen, followed by 30 mg 3 times a week for up to 12 weeks. This resulted in an ORR of 52% in all patients, of which 32% achieved CR. In addition, 6/7 patients with blood involvement had a response. Median time to treatment failure was 12 months (5–32+ months). CMV reactivation occurred in 18% of patients and another 27% of patients had other infections (42). A retrospective study in 39 patients with MF (*n* = 16) and SS (*n* = 23) who received alemtuzumab 30 mg two to three times per week for a median duration of 12 weeks (range 1–35) demonstrated an ORR of 51% in all patients, with a 70% response rate in patients with SS compared to 25% in patients with MF (43). A small number of patients exhibited a durable response. However, severe infectious complications were common. 62% of patients had a grade 3 or higher infectious adverse event resulting in treatment discontinuation in 44% and 2 deaths.

In 2012, alemtuzumab was withdrawn from market in the US and Europe and has since been reintroduced as a treatment for multiple sclerosis with availability on a compassionate use basis in hematologic and oncologic indications.

*Alemtuzumab is an effective therapeutic option, especially in patients with leukemic disease however it's use is limited due to high rates of serious infectious complications*.

### IPH4102

KIR3DL2 negatively modulates immune effector cell functions through binding to HLA Class-1 ligands (44). Normally, KIR3DL2 expression is restricted to minor subpopulations of NK cells and T cells. However, KIR3DL2 has been found to be expressed in all subtypes of CTCL, with the highest prevalence of expression in SS and transformed MF (45). IPH4102 is an anti-KIR3DL2 antibody developed for the treatment of cutaneous T-cell lymphoma (46).

Preliminary results in the SS subset of a phase I dose-escalation and expansion study of IPH4102 in advanced CTCL have been published in abstract form (47). Patients received 10 dose levels up to 10 mg/kg in an accelerated 3 + 3 design with recommended cohort expansion dose of 750 mg. Results demonstrate excellent tolerability and an ORR of 42.9% with median duration of response of 13.8 months and median progression-free survival of 11.7 months (47, 48). Clinical activity was associated with a substantial improvement in quality of life and displayed a favorable safety profile.

Based on these results, the FDA has granted fast track designation to IPH4102 for the treatment of adult patients with relapsed or refractory Sézary syndrome who have received at least two prior systemic therapies. A multi-cohort phase II study in T-cell lymphoma subtypes will be initiated in the first half of 2019 (NCT03902184).

*IPH4102 is an immune NK cell engager with a CTCL specific target that has demonstrated significant promise toward offering a durable and tolerable treatment response*.

### TTI-621

CD47 is expressed on all normal cells and interacts with SIRPα on the surface of myeloid cells, resulting in inhibition of macrophage phagocytosis. CD47 has been found to be overexpressed on cancer cells, suggesting CD47 antagonists may offer a new potential therapy targeted toward malignancies (49). Recent studies of anti-CD47 antibodies have been proven to have therapeutic effects against the tumor microenvironment in CTCL and there are several CD47 antagonists in active phase I clinical trials (50).

TTI-621 is a recombinant fusion protein composed of human SIRPa fused to the Fc receptor of IgG1 (51). In preliminary results of a phase 1 study (52), nine adult patients with mycosis fungoides and Sezary syndrome were treated with a single intratumoral injection of TTI-621 at a dose of 1, 3, or 10mg. TTI-621 was well tolerated with fatigue, chills, and decreased appetite being the main adverse effects and no dose-limiting toxicities noted. All patients experienced declines in tumor size and had decreased circulating Sezary cells with one patient achieving a CR that was ongoing at time of publication (4.2 months). These results suggest that direct antitumoral injection of TTI-621 can elicit local and systemic anti-tumoral activity. There is also an ongoing phase I study of systemic TTI-621 in hematologic malignancies and select solid tumors, including a cohort with CTCL, which is currently recruiting patients (NCT02663518).

*Checkpoint inhibition with anti-CD47 antibodies offer an exciting new potential therapy in many malignancies including CTCL, with evidence of durable and tolerable responses seen with intratumoral administration*.

### AFM13

AFM13 is a bispecific antibody designed to bind to CD30 and to CD16A, a receptor on NK cells that results in NK-mediated killing of tumor cells (53).

A phase 1 study assessed the safety and tolerability of AFM13 in 28 patients with Hodgkin Lymphoma in the relapsed/refractory setting (54). Patients received doses of 0.01–7 mg/kg. Most adverse events were mild to moderate and the maximum tolerated dose was not reached. Of the 26 evaluable patients, 11.5% had a partial remission and 50% had stable disease. There are multiple additional studies ongoing in patients with relapsed/refractory HL.

An investigator-sponsored phase 1/2 study in patients with relapsed/refractory CD30+ CTCL is also ongoing (NCT03192202). Initial results of the first three dose cohorts (9 patients dosed at 1.5–7.0 mg/kg) demonstrated that AFM13 was well tolerated and showed therapeutic activity as a single agent, with an ORR of 44% (4/9) including one CR, three PR, and two patients with SD (55).

*AFM13 is an exciting new immunotherapy that is well-tolerated and in preliminary data has demonstrated activity in patients who have failed standard therapies and have limited to no remaining treatment options*.

### ARGX-110

Overexpression of CD70 has been documented in a variety of cancers, including PTCL and CTCL (56). Signaling mediated by CD70-CD27 is thought to induce proliferation and survival of tumor cells via activation of the NF-κB pathway. In addition, T-reg cells expressing CD27 can be activated by CD70 cells and contribute to an immunosuppressive tumor microenvironment (57, 58). ARGX-110 is a human, defucosylated IgG_1_ monoclonal antibody that binds to CD70 and blocks signaling mediated by its interaction with CD27. In addition to interrupting CD70-CD27 signaling, ARGX-110 also induces antibody-dependent cellular cytotoxicity in CD70+ tumor cells (58). A phase 1b trial focused on the use of ARGX-110 in T-cell malignancies is currently ongoing (NCT01813539). Initial results have demonstrated overexpression of CD70 in 28/26 patients. ARGX-110 has been administered to 16 patients with CTCL with disease control and response seen in 9 patients, including 3 PR. Treatment has been well tolerated with no grade 3 toxicities noted to date (59, 60).

*ARG-110X offers an attractive potential therapy with early evidence of anti-tumor activity that is well tolerated*.

## Conclusion

There is a significant need for new systemic treatments that offer durable and tolerable responses in patients with CTCL. Antibody-directed therapies offer significant potential in these incurable illnesses with the potential for lasting responses and manageable toxicity. The recent approval of the CD30 directed antibody-drug conjugate brentuximab vedotin and anti-CCR4 antibody mogamulizumab represent ground-breaking developments in this process. There are additional promising modalities currently under study including anti-PD-1/PD-L1 therapy, KIR3DL2 inhibition, and novel IL-2 fusion toxins, amongst others (see Table 2). These advances have been based on extensive work understanding the molecular characteristics, intracellular signaling pathways, and interaction with the tumor microenvironment in CTCL, which continue to offer new targets of potential treatment. Toxicities remain a prominent concern with these therapies and will need to be closely examined in the evaluation of novel agents and combination regimens.

**Table 2:** Experimental antibody therapies.

**Class**	**Drug**	**ORR**	**# of patients**	**Study phase**
IL-2 fusion protein	E777	29% 38%	17 13	Phase I (39) Phase I (40)
Anti-CD52 Mab	Alemtuzumab	52% 51%	22 39	Phase II (42) Retrospective (43)
Anti-KIR3DL2 Mab	IPH4102	43%	13	Phase I (47)
Anti-PD-1/PD-L1 Mab	Pembrolizumab Nivolumab	38% 15%	24 13	Phase II (36) Phase I (38)
Anti-CD47 Mab	TTI-621	Not reported	9	Phase I (52)
Bispecific CD30-CD16 ab	AFM13	44%	9	Phase I (55)
Anti-CD70 Mab	ARGX-110	56%	16	Phase I (60)

## Author Contributions

All authors listed have made a substantial, direct and intellectual contribution to the work, and approved it for publication.

### Conflict of Interest Statement

AS: Consultation: Seattle Genetics and Kyowa Kirin. Research support: Seattle Genetics, Affimed. The remaining author declares that the research was conducted in the absence of any commercial or financial relationships that could be construed as a potential conflict of interest.
